# Suspected Lonely Mouse Syndrome as a Cage Effect in a Drug Safety Study

**DOI:** 10.1155/2018/9562803

**Published:** 2018-05-09

**Authors:** Xiaobu Ye, MariaLisa Itzoe, Rachel Sarabia-Estrada, Louis DeTolla, Betty M. Tyler, Michael Guarnieri

**Affiliations:** ^1^Department of Neurological Surgery, Johns Hopkins School of Medicine, Baltimore, MD, USA; ^2^School of Medicine, Departments of Pathology, Medicine, Epidemiology and Public Health and the Program of Comparative Medicine, University of Maryland, Baltimore, MD, USA

## Abstract

Studies have demonstrated that buprenorphine, a front line drug for veterinary analgesia, may alleviate symptoms of chronic pain. A cage side observation protocol was used to record behavioral signs in a mouse clinical trial of extended release buprenorphine. A retrospective review of the observations for signs of pain and stress revealed that mice given a fivefold overdose of buprenorphine (16.25 mg/kg) showed lethargy and facial signs associated with stress. However, similar signs were observed in the drug-free control mice as early as Day 3 of single-cage housing. This appears to be the first report of cage effects in a clinical trial for a veterinary drug.

## 1. Introduction

Centers for veterinary medicine at the Food and Drug Administration (FDA) in the US and the European Medicines Agency provide guidance documents for clinical trials to evaluate the safety and efficacy of new veterinary pharmaceuticals [1, 2]. The documents, also known as Target Animal Safety (TAS) guidelines, recommend comprehensive literature reviews and protocol-driven laboratory studies including histopathology examinations and behavioral observations in trials using excess doses of drug. Laboratory tests and observations should focus on anticipated side effects, that is, those associated with the class of drugs being tested and also for paradoxical and unanticipated side effects. Evaluations should be blinded. Single caging is advised to avoid inadvertent drug transfers. In addition, because each cage can be treated as a unit for statistical purposes, single-cage studies reduce the number of animals used.

We conducted TAS trials to examine the safety of a lipid-bound buprenorphine analgesic in mice [3, 4]. During preliminary meetings with the FDA's Center for Veterinary Medicine questions were raised about behavioral observations of mice in single cages. Mice are social animals. Cage side observations for behavioral anomalies consider signs of aggression, cowering, and avoidance in relation to interactions with cage mates. Because opiates affect behavior through multiple pathways, early and modest signs of neurological changes could be masked. Nonetheless, advice to use a single-cage protocol was consistent. In our trials, single caging would last for a maximum of 12 days. Behavioral analyses in many species have demonstrated isolation effects, yet the effects in wild type mice generally depend on isolation of several weeks [5–8] and may be strain dependent [9, 10].

Our daily clinical observations generated reports for each mouse in the trials. As the drug was intended for postsurgical analgesia, the observations recorded physical signs and behaviors that are consensus indicators of pain and distress. In addition to observations focused on the surgical site, our studies collected approximately 20,000 data points regarding the behavior of mice treated with drug and their drug-negative controls. Because the trials involved a large dose of an opiate, we expected to measure modest and transient signs of nausea, lethargy, and stress, which are common side effects of opiate therapy in animals and humans. These signs were observed in mice treated with 16.25 mg/kg of drug, a fivefold excess of the intended dose, and will be reported elsewhere. Surprisingly, an analysis of the data from the control mice demonstrated significant signs of lethargy and stress in the drug-free animals. The signs increased in the days following surgery. Thus, we believe that the behaviors can be attributed to the stress of social isolation and provide evidence that the lonely mouse phenomenon may play a previously unexpected role in similar short-term drug trials.

## 2. Methods

Studies were approved by University Institutional Animal Care and Use Committee (IACUC). The IACUC protocol complies with the National Research Council's Guide for the Care and Use of Laboratory Animals and fulfills the requirements of the Association for the Assessment and Accreditation of Laboratory Animal Care, International Program. The study was conducted at University Department of Molecular and Comparative Pathobiology. Health surveillance and detection of pathogen contaminants were conducted by a soiled-bedding sentinel system. Male and female BALB/cAnNCrl mice (6–8 weeks old; weighing 20–22 g) were obtained from Charles River Laboratories (Wilmington, MA). Mice were inspected for general health conditions before being housed at a population of 4-5 mice per cage in Smart Bio-Pak cages (Allentown, NJ) with Tek-Fresh bedding (Harlan, Madison, WI) and were allowed free access to Teklad Global Rodent Diet chow (Harlan, Madison, WI) and defined water. Mice housed 3-4 per cage were held for approximately 10 days prior to the start of the experiment. Mice were weighed prior to assignment to the drug or control groups to ensure the weight of each mouse was within 10% of the average group weight at the start of the study. Throughout the duration of the study, mice were housed individually. Cages were changed daily to prevent buprenorphine redosing by coprophagy.

The study design was based on TAS guidelines for assessing the safety of veterinary pharmaceutical products [11]. Because a previous study demonstrated the safety and unremarkable behavioral changes of 1-, 3-, and 5-fold doses of a powdered form of the drug [4], a 5-fold excess dose was utilized to examine safety in this study.

Eight male and eight female mice per group were used in Trial 1 comparing a control (0.0 mg/kg) and 5-fold dose (16.25 mg/kg) challenge. Eight male and eight female mice per group were used in Trial 2 comparing control and 5-fold doses, repeated at three 4-day intervals. As shown in Table 1, at the midpoints of both trials, either at Day 2 of Trial 1 or at Day 6 of Trial 2, half of the mice (4 of each group) were weighed, euthanized, and exsanguinated for hematology and clinical chemistry analyses. At the endpoints of Trials 1 and 2, Day 4, and Day 12, respectively, the remaining mice were euthanized to measure body weight, hematology, clinical chemistries, and anatomic pathology. Mice were euthanized by carbon dioxide asphyxiation, exsanguination by cardiac puncture and a thoracotomy. Body weights, hematology, clinical chemistry, gross pathology, and histopathology parameters evaluated in Trials 1 and 2 have been previously reported [3].

The cholesterol-buprenorphine drug powder was supplied by Animalgesic Laboratories Inc. (Millersville MD). The drug powder contained USP (United States Pharmacopeia) grade buprenorphine HCl (Noramco, Wilmington DE), cholesterol, and glycerol tristearate, (Sigma, St Louis MO). Drug preparations were verified for purity and content by AAI Pharma (Wilmington NC). Negative control, drug-free powder was prepared by tumble blending a mixture of cholesterol and glycerol tristearate (96/4, w/w) for 48 hours at 5°C. Injectable suspensions of drug powder and the control were prepared by suspending 80 mg of powder per mL of medium chain triglyceride (MCT) oil (Miglyol 812, from Sasol, Hamburg Germany) followed by brief shaking to make a homogeneous suspension. Suspensions were generally prepared within 1-2 days of use and stored at 2–8°C. A single (1x) dose consisted of a 0.05 mL drug suspension. One mL syringes with 1 inch 20 gauge needles were used to inject suspensions (described below) of cholesterol-triglyceride-buprenorphine powder and the negative control, cholesterol-triglyceride control powder. The surgical procedure was based on the procedure used to implant Alzet miniosmotic pumps in mice and rats. A video of the subcutaneous implantation procedure, which is briefly described below, is available at the Alzet website [12].

Mice were given intraperitoneal (IP) anesthesia with a solution containing 8.4 mg/mL (65 mg/kg) ketamine, 0.84 mg/mL (0.65 mg/kg) xylazine, and 4.75% ethanol in saline. The dose of anesthesia was 0.15 mL/20 g mouse. When anesthesia was established, approximately 1 cm^2^ of middorsal skin was shaved, washed with ethanol, and then coated with povidone-iodine solution. Mice were transferred to a procedural table that was cleaned with 70% ethanol solution and covered with a clean disposable towel. A sterile disposable number 10 blade was used to make a 4-5 mm incision through the skin only. Bleeding, if any, was controlled with sterile gauze and light pressure. Sterile forceps were used to separate the skin and to create approximately a 2 × 4 cm subcutaneous pocket. The skin was then apposed and stapled with 9 mm Autoclips (Kent Scientific, Torrington CT). After the skin was stapled, mice were injected with either the drug or control suspension (0.25 mL/mouse) into the interscapular subcutis. All mice in the study were treated to this “sham surgical procedure.” After the procedure, mice were moved to a holding cage. This cage contained a 37°C heating pad covered with a clean disposable towel. When the mouse regained consciousness, as demonstrated by movement and the absence of signs of distress, which included but were not limited to abnormal paw movements, efforts to scratch the incision site, and cowering, each mouse was placed individually into a clean cage.

The two trials had a different number of observation days: 4 days in Trial 1 and 5 days in Trial 2. Behavioral data per mouse were collected in the morning, between 8-9 am, and evenings, between 5-6 pm. The cage side observations were conducted and recorded by the same female veterinarian, blind to the treatment groups. To quantify this data, FDA validated observation forms were used. The forms were designed for the entry of numerical grading of the extent to which pain or distress was present across nine parameters: mouse respiration, nasal/skin appearance, fur appearance, motor activity, ocular activity (closed or open eyes), behavior suggesting stress (i.e., aggressiveness), presence of tremors, and surgical site erythema, edema, or infection (swelling, pus, and exudate). Ratings were made on scales ranging from 1 to 3 or from 1 to 6, depending on the parameter. Higher numbers indicated more severe signs of pain/distress. The ocular score was recorded using a scale of 1 to 5: (1) no observed abnormalities (NOA); (2) squinting; (3) eyes closed; (4) crusty secretions; and (5) porphyrin stain. The motor activity was recorded using a scale from 1 to 4: (1) NOA; (2) rapid darting; (3) hunched/lethargic; and (4) hunched/motionless. In addition, a “yes/no” score was given for an assessment of the general condition of each mouse and its cage. Space on the forms was also available for comment. Observations of the following were recorded twice daily: motor activity, ocular signs, fur appearance, and general conditions. The daily observation form for each mouse hereinafter is referred to as the mouse chart.

Outcome of the experiments on ocular and motor actives were described as ordinal scores. Data were summarized as frequency and percentage. The possible natural variability of the ocular score and motor activity with respect to trial, gender, time (morning versus afternoon), and day were assessed using data from the control group. Chi-square test was used for group comparisons and treatment comparison between the drug and the control. Subgroup comparisons were performed due to statistically significant confounding factors which were identified using the control data. All *p* values were reported as 2-sided, and all analyses were conducted using SAS software, version 9.2 (SAS Institute, Cary, NC).

## 3. Results

The 272 charts from the drug-free control male and female mice in Trial 1 and in Trial 2 were analyzed to examine whether elements in the experimental design, including single-cage housing, were potentially confounding factors. The analyses of the control data revealed a significant difference in the observation of ocular scores between the two trials. As shown in Table 2, significantly more mice in the drug-free control group were observed with closed eyes in Trial 2 (27.8%) than Trial 1 (13.6%). The significant difference of the distribution on ocular score between the two trials (*p* = 0.0176) among untreated mice may indicate variation in conducting the trials. Gender difference was observed in motor activity behavior. The distribution of the motor activity was significantly different between the male and female mice (*p* = 0.0018) of the drug-free controls (Table 3). More female mice (30.0%) were observed with rapid, darting movements compared to the male group (10.8%) and higher percentage of male mice at higher score of hunched posture than the female mice. Gender differences were not seen in ocular scores of the control mice. Our experiments also show significant time effect on ocular score and motor activity on mice without drug interventions (*p* = 0.0135 and *p* = 0.0076, resp.). As seen in Table 4, over the study's duration, mice within the drug-free control groups from both trials exhibited increasing signs of lethargy and stress (Day 1 versus Day 5).

## 4. Discussion

The present study demonstrates a potentially confounding variable in studies using TAS protocols for assessing drug safety in mice. Single-cage studies can introduce a lonely mouse syndrome. Stress avoidance should be optimized in every animal model. However, lonely mouse models have provided tools to investigate behavioral phenomenon including autonomic stress and negative emotional states. Recent studies by Furuhashi and Sakamoto and by Matthews et al. [13, 14] have outlined potential neural mechanisms. The latter group used genetically engineered mice to demonstrate that the initial signs of stress can be detected within 48 hours by optogenetic activation of dopamine neurons. Moreover, single-cage protocols have compelling advantages in studies involving surgically treated animals. Single-cage protocols eliminate complications that arise from mice scratching or gnawing at the wounds of cage mates and from redosing via coprophagy. It seems likely that enrichment tools could be utilized to minimize stress in these models [15–17].

The cage side observation tool used in the present study appears to provide a sensitive and specific tool to detect mouse stress. More research is needed to confirm that our results demonstrating behavioral studies using a numerical scoring system across five independent variables can provide an early diagnosis for stress in mice: (1) changes in body weight; (2) external appearance; (3) measurable clinical signs; (4) unprovoked behavior; and (5) behavioral responses to external stimuli. Since first proposed more than 30 years ago by Morton and Griffiths, the use of a numerical scoring system has become a widely accepted method of objectively assessing pain and distress in laboratory animals [18, 19]. Yet, to our knowledge, the system has not been validated, and it seems intuitive that automated recording systems that compared to the cage side observation protocol used in the present assesses behavior through the light and dark cycle would provide a more sensitive and specific tool [13, 20–22]. Nonetheless, comparisons of behavioral data obtained by cage side observation protocols, especially those conducted only during light cycles, with automated recording systems monitoring light and dark cycle activity are scarce.

## Figures and Tables

**Table 1:** Experimental design displaying the number of daily observations and harvest schedule for the single dose (Trial 1) and repeat dose (Trial 2) studies.

	Trial day								

	Single dose: Trial 1								
Number of mice	Days 1	2	3	4	5				
Per dose 16♂, 16♀	32								
per harvest		16^*∗*^		16^*∗∗*^					
Charts AM		32	16	16					
Charts PM	32	16	16	16					
	Repeat dose: Trial 2								
Number of mice	Days 1	2 to 3	4	5	6	7	8	9 to 11	12
Per dose 16♂, 16♀	32		32				16		
Per harvest					16^*∗*^				16^*∗∗*^
Charts AM		32	32	32	32	16	16	16	
Charts PM	32	32	32	32	16	16	16	16	

^*∗*^Weight, hematology, and clinical chemistry; ^*∗∗*^weight, hematology, clinical chemistry, coagulation panel, organ weight, and histopathology.

**Table 2:** Variability of ocular scores for drug-free (Control) animals in Trials 1 and 2.

Ocular score^*∗*^	Trial 1	Trial 2
NOA:^*∗∗*^ number (%)	82 (85.4)	104 (72.2)
Eyes closed: number (%)	13 (13.6)	40 (27.8)
Crusty secretions: number (%)	1 (1.0)	0 (0)

^*∗*^ *p* = 0.0176; ^*∗∗*^no observed abnormalities.

**Table 3:** Significant gender difference in motor activity of drug-free (control) animals.

Motor score:^*∗*^	Male (%)	Female (%)
NOA: number (%)	85 (70.8)	63 (52.5)
Rapid, darting: number (%)	13 (10.8)	36 (30.0)
Hunched, lethargic: number (%)	2 (1.7)	4 (3.3)
Hunched, motionless: number (%)	20 (16.7)	17 (14.2)

^**∗**^ *p* = 0.0018.

**Table 4:** Frequency and percentage distributions of signs of stress increase in drug-free (control) animals over a 5-day time period.

Ocular score^*∗*^	Day 1	Day 2	Day 3	Day 4	Day 5
NOA: number (%)	26 (81.3)	55 (85.9)	45 (80.4)	38 (79.2)	22 (55.0)
Eyes closed: number (%)	6 (18.7)	9 (14.1)	11 (19.6)	9 (18.7)	18 (45.0)
Crusty secretions: number (%)	0 (0.0)	0 (0.0)	(0.0)	1 (2.1)	0 (0.0)

Motor score^*∗∗*^	Day 1	Day 2	Day 3	Day 4	Day 5

NOA: number (%)	17 (53.2)	46 (71.9)	35 (62.5)	31 (64.6)	19 (47.5)
Rapid, darting: number (%)	11 (34.4)	9 (14.1)	11 (19.6)	10 (20.8)	8 (20.0)
Hunched, lethargic: number (%)	3 (9.4)	1 (1.6)	2 (3.6)	0 (0.0)	0 (0.0)
Hunched, motionless: number (%)	1 (3.1)	8 (12.5)	8 (14.3)	7 (14.6)	13 (32.5)

^**∗**^ *p* = 0.0135; ^**∗∗**^*p* = 0.0076.
